# Severe *Plasmodium vivax* Malaria Presenting With Circulatory Shock and Coagulation Abnormalities: A Case Report

**DOI:** 10.1002/ccr3.73867

**Published:** 2026-11-03

**Authors:** Bushra Majid, Azmat Ullah Jan, Fatima Aman, Ahmad Ali, Waseem Abbas, Asim Shah, Ali Shir Erkin, Suleman Khan, Aizaz Anwar Khalid

**Affiliations:** ^1^ Rehman Medical Institute Peshawar Pakistan; ^2^ Peshawar Medical College Peshawar Pakistan; ^3^ Khyber Medical College Peshawar Pakistan; ^4^ Kabul University of Medical Sciences (KUMS) Kabul Afghanistan

**Keywords:** circulatory shock, coagulation activation, hypoxemia, *Plasmodium vivax*, severe malaria, thrombocytopenia

## Abstract

Severe *Plasmodium vivax* malaria may cause vasopressor‐requiring shock, profound thrombocytopenia, jaundice, coagulation activation, and transient cardiopulmonary abnormalities. Early parasite confirmation and close monitoring for evolving organ dysfunction are essential.

## 1 Introduction

Malaria caused by Plasmodium species can be life‐threatening. Although *P. vivax* was traditionally considered less virulent than *P. falciparum*, severe disease is recognized and may include thrombocytopenia, jaundice, anemia, respiratory complications, kidney injury, neurologic dysfunction, and shock [1, 2, 3, 4].

Severe vivax malaria has been reported across endemic regions, including Pakistan; estimates vary because studies use heterogeneous definitions and often involve hospitalized patients [2, 3, 4].

Proposed mechanisms include inflammatory activation, endothelial dysfunction, altered microvascular physiology, and splenic retention of infected erythrocytes [5]. Coagulation abnormalities, including malaria‐associated disseminated intravascular coagulation (DIC), have also been reported [6, 7]. Diagnosis relies on parasite detection and careful assessment of organ dysfunction [8].

This case illustrates the coexistence of vasopressor‐requiring shock, profound thrombocytopenia, jaundice, marked D‐dimer elevation, and transient hypoxemia with troponin elevation in an adult with repeated blood‐smear identification of *P. vivax* . The cardiopulmonary event and coagulation findings required cautious interpretation because neither a specific cardiac/pulmonary cause nor overt DIC was established.

## 2 Case Presentation

A previously healthy 37‐year‐old man presented after 5 days of intermittent high‐grade fever, 3 days of loose stools, and worsening lethargy. Before admission, hemoglobin was 13.3 g/dL, leukocytes 3.0 × 10^9^/L, platelets 12.1 × 10^9^/L, urea 145 mg/dL, BUN 59 mg/dL, and creatinine 0.91 mg/dL. Dengue NS1 was negative and a malaria smear was positive for *P. vivax* .

At presentation, he was toxic and lethargic but arousable (GCS 14/15); blood pressure was 75/46 mmHg, pulse 86/min, respiratory rate 12/min, temperature 98°F, oxygen saturation 96%, and glucose 60 mg/dL. Examination showed deep scleral jaundice and mild splenomegaly without visible bleeding or bruising; initial chest and cardiovascular examinations were unremarkable.

He received 3 L of intravenous normal saline, four ampules of 25% dextrose, and noradrenaline for persistent hypotension (charted rate 5 mL/h). The medication chart recorded oral artemether/lumefantrine 120 mg twice daily from 18 to 20 August 2026, empirical piperacillin/tazobactam 4.5 g while sepsis was considered, and one 100‐mg hydrocortisone dose. No parenteral artesunate was identified in the supplied chart.

Emergency‐department tests showed hemoglobin 11.9 g/dL, leukocytes 5.89 × 10^9^/L, platelets 26 × 10^9^/L, creatinine 1.10 mg/dL, urea 62 mg/dL, eGFR 85 mL/min/1.73 m^2^, potassium 3.3 mmol/L, bilirubin 6.2 mg/dL, albumin 2.5 g/dL, LDH 1007 U/L, and D‐dimer 15,448 ng/mL (reference 0–475).

Persistent hypotension prompted ICU transfer and continued noradrenaline and intravenous fluids. Stool examination and occult blood were negative. Urinalysis showed protein 2+, bilirubin 3+, urobilinogen 2+, and 15–20 red cells/high‐power field, with 1–2 pus cells/high‐power field and negative nitrites.

Repeated peripheral smears showed trophozoites and gametocytes morphologically consistent with *P. vivax* . A representative peripheral blood smear demonstrating *P. vivax* forms is shown in Figure 1. The ICT malaria antigen test was not detected; no species‐specific PCR was performed, so a mixed infection cannot be excluded with certainty. Abdominal ultrasonography was reported as normal.

**FIGURE 1:**
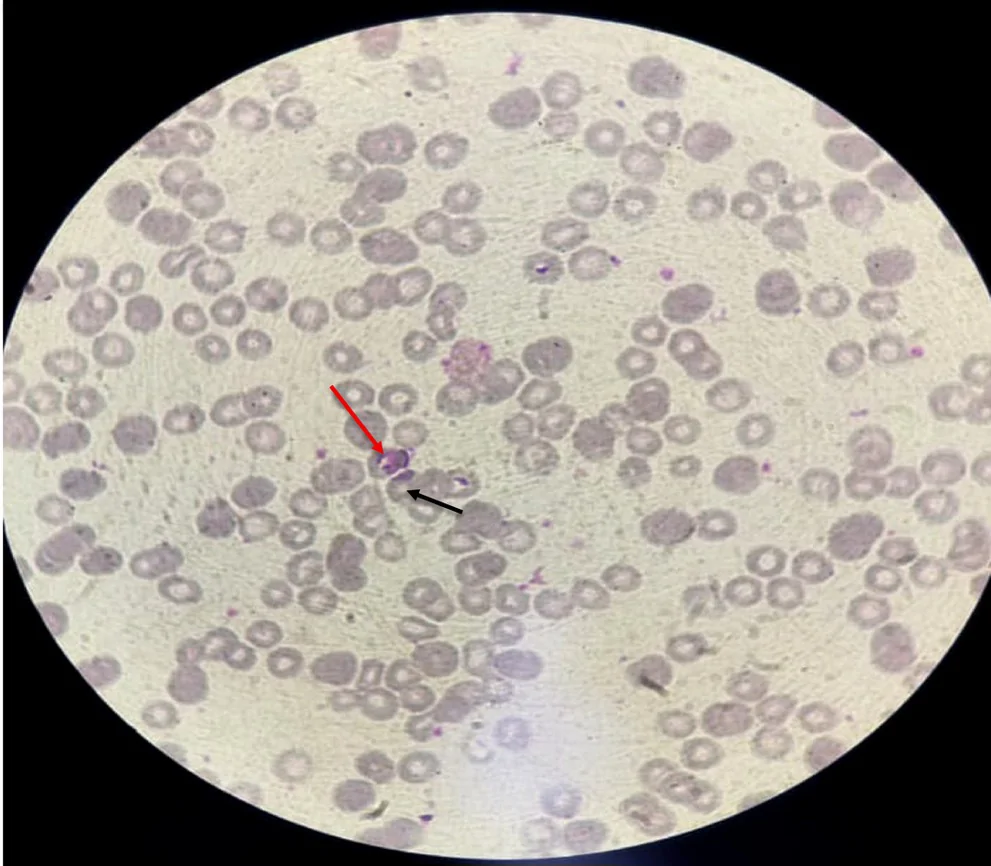
Peripheral blood smear demonstrating *Plasmodium vivax* forms. The red arrow indicates a gametocyte; the black arrow indicates a ring‐form trophozoite.

After approximately 24 h, GCS improved to 15/15 and D‐dimer decreased to 8936. Repeat tests showed PT 10.4 s, INR 0.98, APTT 35.5 s, bleeding time 7 min 45 s, clotting time 8 min 30 s, and fibrinogen 414.1 mg/dL (not reduced).

On day 3, sudden dyspnea and chest pain developed with oxygen saturation falling to 83%–85%; high‐sensitivity troponin‐I was 306 ng/L. ECG showed no acute changes and echocardiography found no major structural or regional wall‐motion abnormality. He received oxygen at 4 L/min and electrolyte replacement. CT pulmonary angiography and cardiac MRI were not documented, so pulmonary embolism, myocarditis, and other causes were not definitively excluded.

By day 4, he was afebrile for about 24 h, blood pressure was 110–120/65–70 mmHg, and noradrenaline was discontinued. Blood culture reported coagulase‐negative staphylococci as a skin contaminant; urine culture showed no growth. He recovered sufficiently to mobilize independently and tolerate a regular diet before discharge. Later platelet values rose to 419 × 10^9^/L. G6PD activity was 11 U/g hemoglobin, and primaquine 7.5 mg twice daily for 14 days was prescribed for radical cure. Post‐discharge follow‐up duration and laboratory results were unavailable.

## 3 Differential Diagnosis

The main differential diagnoses and supporting evidence are summarized in Table 1.

**TABLE 1:** Differential diagnoses considered during the acute presentation.

Diagnosis	Why considered	Supporting evidence	Status/interpretation
*Severe P. vivax malaria*	Fever, thrombocytopenia, jaundice, splenomegaly, shock.	*Repeated smears showed P. vivax forms; hypotension required vasopressor support*.	Microscopy confirmed parasitemia; fits clinical syndrome.
Bacterial sepsis/septic shock	Toxic appearance and hypotension persisting after fluids.	Persistent shock prompted ICU care and empirical antibiotics.	Blood culture deemed a skin contaminant; urine culture negative; no source.
Dengue	Thrombocytopenia and shock can occur in severe dengue.	Severe thrombocytopenia and hypotension.	Dengue NS1 negative; malaria smear positive.
DIC/malaria‐associated coagulopathy	Severe thrombocytopenia and markedly high D‐dimer.	D‐dimer 15,448, decreasing to 8936; mild APTT prolongation.	PT/INR near normal, fibrinogen not low; DIC unconfirmed.
Hypovolemic shock from diarrhea	Loose stools could contribute to fluid depletion.	Received approximately 3 L IV saline.	Hypotension persisted; dehydration not established as the main cause.
Pulmonary embolic/cardiac event	Day‐3 dyspnea, chest pain, hypoxemia, and troponin elevation.	SpO_2_ 83%–85%; hs‐troponin‐I 306 ng/L.	ECG/echo nondiagnostic; no CT angiography or cardiac MRI; cause unresolved.

## 4 Investigations

Selected laboratory and diagnostic findings by time point are summarized in Table 2.

**TABLE 2:** Key investigations by time point.

Time point	Investigation	Result	Reference interval/unit	Clinical significance
Before admission	CBC/renal profile	Hb 13.3 g/dL; TLC 3.0 × 10^9^/L; platelets 12.1 × 10^9^/L; urea 145 mg/dL; BUN 59 mg/dL; creatinine 0.91 mg/dL	As reported	Thrombocytopenia, leukopenia, azotemia.
Before admission	Dengue/malaria	*Dengue NS1 negative; smear positive for P. vivax*	Qualitative	Supports malaria.
Emergency department	CBC	Hb 11.9 g/dL; TLC 5.89 × 10^9^/L; platelets 26 × 10^9^/L	Platelets 150–400 × 10^9^/L	Severe thrombocytopenia.
Emergency department	Renal/metabolic	Creatinine 1.10 mg/dL; urea 62 mg/dL; eGFR 85 mL/min/1.73 m^2^; K 3.3 mmol/L; glucose 60 mg/dL	As reported/standard units	Improved azotemia; treated hypoglycemia/hypokalemia.
Emergency department	Liver/tissue injury	Bilirubin 6.2 mg/dL; albumin 2.5 g/dL; LDH 1007 U/L	LDH 135–225 U/L	Hyperbilirubinemia; elevated LDH.
Emergency department	Coagulation	PT 10.1 s; INR 1.0; APTT 29.9 s; D‐dimer 15,448 ng/mL	PT 10–14 s; INR 0.9–1.3; D‐dimer 0–475 ng/mL	High D‐dimer; PT/INR preserved.
ICU, ~24 h	Coagulation trend/fibrinogen	D‐dimer 8936; PT 10.4 s; INR 0.98; APTT 35.5 s; fibrinogen 414.1 mg/dL	APTT 26–34 s; fibrinogen 200–400 mg/dL	Falling D‐dimer; fibrinogen not reduced.
ICU	Smear/urinalysis	*P. vivax trophozoites and gametocytes; urine protein 2+, bilirubin 3+, RBC 15–20/HPF*	Qualitative/per laboratory	Smear supports diagnosis; urinary abnormalities.
Day 3	Cardiac/respiratory	SpO_2_ 83%–85%; hs‐troponin‐I 306 ng/L; ECG without acute changes; echo not significant	Troponin 0–33 ng/L	Hypoxemia/troponin elevation; cause unconfirmed.
Before discharge	G6PD	11 U/g hemoglobin	As reported	Measured before primaquine prescription.

## 5 Treatment

Documented interventions are summarized in Table 3. WHO guidance recommends parenteral artesunate for severe malaria until oral treatment can be tolerated, followed by completion of an appropriate oral regimen [8]. The available chart documented oral artemether/lumefantrine but no parenteral artesunate; treatment is reported as documented rather than retrospectively inferred. Primaquine was prescribed after G6PD testing for radical cure [8].

**TABLE 3:** Therapeutic interventions documented in the case record.

Intervention	Dose	Route/frequency	Duration/timing	Notes
Artemether/lumefantrine	120 mg as charted	Oral; twice daily	Entries span 18–20 Aug 2026	Exact formulation not inferred; parenteral artesunate absent from chart.
Normal saline	2 L bolus +1 L	IV	Acute resuscitation	For persistent hypotension.
Dextrose 25%	4 ampules	IV	Acute correction	For glucose 60 mg/dL.
Noradrenaline	5 mL/h as charted	Continuous IV infusion	Stopped on day 4	Concentration/weight‐based dose unavailable.
Piperacillin/tazobactam	4.5 g	As recorded in chart	Empirical	Given while bacterial sepsis was considered.
Hydrocortisone	100 mg	Single documented dose; route unclear	Emergency department	For persistent hypotension; no further course documented.
Oxygen	4 L/min	Supplemental oxygen	Day 3	For desaturation to 83%–85%.
Electrolyte replacement	Calcium and potassium	Not specified	Day 3	For low ionized calcium and potassium.
Primaquine	7.5 mg twice daily	Oral	14 days; prescribed at follow‐up	After G6PD testing; radical cure.

*Note:* This distinction is important: the favorable outcome followed the documented antimalarial and supportive care, but it does not establish the contribution of any individual intervention.

## 6 Follow‐Up and Outcome

GCS improved from 14/15 to 15/15 within 24 h. Blood pressure stabilized by day 4, allowing noradrenaline discontinuation, and D‐dimer fell from 15,448 to 8936. The day‐3 hypoxemia and chest pain resolved with oxygen and supportive care, without a confirmed cardiopulmonary diagnosis.

Platelets subsequently recovered, with a later recorded value of 419 × 10^9^/L. The patient became afebrile, transferred from ICU to the ward, and was discharged after regaining independent mobility and oral intake. No adverse drug reaction was documented; long‐term clinical status and malaria recurrence were not available.

## 7 Final Diagnosis

The final diagnosis was severe *P. vivax* malaria with circulatory shock, profound thrombocytopenia, jaundice, marked coagulation activation, and transient hypoxemia with troponin elevation. Repeated microscopy supported *P. vivax* , but mixed infection could not be absolutely excluded because molecular testing was not performed.

Dengue was less likely after negative NS1 testing. Cultures did not establish a bacterial source. Near‐normal PT/INR and non‐reduced fibrinogen did not establish overt DIC, and the cause of the day‐3 cardiopulmonary event remained uncertain.

## 8 Discussion

This case illustrates life‐threatening multisystem *P. vivax* malaria in a previously healthy adult, featuring vasopressor‐dependent shock, profound thrombocytopenia, jaundice, marked coagulation activation, and transient hypoxemia with troponin elevation. Severe vivax malaria is well recognized despite its historical reputation as less virulent than *P. falciparum*; reported complications include respiratory, hematologic, neurologic, renal, and other organ dysfunction [1, 2, 3, 4, 9].

Studies from Pakistan and India describe severe vivax disease, including thrombocytopenia and jaundice, although comparisons are limited by heterogeneous settings and case definitions [3, 4, 9]. Reports also document pulmonary and cerebral complications and substantial multisystem morbidity [10, 11, 12].

Repeated microscopy supported *P. vivax* despite a negative ICT antigen result. Because PCR was not performed, mixed infection cannot be excluded completely. Microscopy and rapid tests have sensitivity limitations, particularly with low parasite density [13].

Coagulation activation was a central issue: D‐dimer was 15,448 and fell to 8936, while PT/INR stayed near normal and fibrinogen was not reduced. Coagulation activation and non‐overt DIC have been described in vivax malaria, but estimates of DIC vary with diagnostic criteria and study populations [6, 7]. The available results support marked coagulation activation, not confirmed overt DIC.

The day‐3 event comprised sudden dyspnea, chest pain, hypoxemia, and troponin elevation. ECG and echocardiography did not establish a cause, and CT pulmonary angiography or cardiac MRI was not documented. Pulmonary embolism, myocarditis, acute coronary syndrome, and other causes therefore remain unexcluded; the event is best described as transient hypoxemia with biochemical myocardial injury [14].

The chart documents oral artemether/lumefantrine, fluid resuscitation, noradrenaline, dextrose, empirical antibiotics, a single hydrocortisone dose, oxygen, and electrolyte replacement. WHO guidance recommends parenteral artesunate for severe malaria [8], but none was identified in the supplied chart. This report describes the recorded care and does not infer the rationale for undocumented decisions or attribute recovery to a single intervention.

Strengths include repeated microscopy, documented vasopressor‐dependent shock, serial coagulation results, and the day‐3 cardiopulmonary event. Limitations include absent molecular confirmation and parasite‐density quantification, incomplete evaluation of the cardiopulmonary event, and limited post‐discharge follow‐up. These uncertainties support transparent case reporting [15].

Clinically, *P. vivax* should not be assumed to be uncomplicated when shock, severe thrombocytopenia, jaundice, hypoglycemia, or respiratory/cardiac abnormalities occur. A high D‐dimer warrants assessment of the full coagulation profile rather than automatic diagnosis of DIC. Rapid antimalarial treatment and close monitoring for evolving organ dysfunction remain essential [8].

## 9 Conclusion

Severe *P. vivax* malaria may cause shock, profound thrombocytopenia, jaundice, coagulation activation, and transient cardiopulmonary abnormalities. In this case, repeated microscopy supported the diagnosis; overt DIC was not established, and the cause of the hypoxemic/troponin‐positive event remained uncertain. Early recognition, antimalarial therapy, and intensive monitoring accompanied clinical recovery.

## Author Contributions

**Bushra Majid:** methodology, validation, writing – original draft. **Azmat Ullah Jan:** conceptualization, resources. **Fatima Aman:** conceptualization, resources. **Ahmad Ali:** writing – review and editing, methodology, writing – original draft. **Waseem Abbas:** investigation, writing – review and editing, supervision. **Asim Shah:** conceptualization. **Ali Shir Erkin:** validation, resources. **Suleman Khan:** supervision. **Aizaz Anwar Khalid:** supervision.

## Funding

The authors have nothing to report.

## Ethics Statement

This case report was conducted in accordance with the principles of the Declaration of Rehman Medical Institute, Peshawar, Pakistan. Written informed consent was obtained from the patient for publication of this case report and any accompanying images. No institutional ethics committee approval was required for this individual case report; however, patient confidentiality and anonymity were maintained throughout.

## Consent

Written informed consent was obtained from the patient for publication of this case report, including all clinical details and images, in accordance with the journal's policies.

## Conflicts of Interest

The authors declare no conflicts of interest.

## Data Availability

The data that support the findings of this study are available from the corresponding author upon reasonable request.
